# Physical activity in Hodgkin's lymphoma survivors with and without chronic fatigue compared with the general population – a cross-sectional study

**DOI:** 10.1186/1471-2407-7-210

**Published:** 2007-11-12

**Authors:** Line M Oldervoll, Jon H Loge, Stein Kaasa, Stian Lydersen, Marianne J Hjermstad, Lene Thorsen, Harald Holte, Anne B Jacobsen, Sophie D Fosså

**Affiliations:** 1 Department of Cancer Research & Molecular Medicine, Faculty of Medicine, the Norwegian University of Science and Technology (NTNU), Trondheim, Norway; 2 Palliative Medicine Unit, Department of Oncology, Trondheim University Hospital, Norway; 3 Department of Clinical Cancer Research, Rikshospitalet-Radiumhospitalet Medical Center, Faculty of Medicine, University of Oslo, Norway; 4 Department of Oncology, Ullevaal University Hospital, Oslo, Norway; 5 Unit for Applied Clinical Research, NTNU, Trondheim, Norway; 6 Department of Medical Oncology, Cancer Clinic, Rikshospitalet-Radiumhospitalet Medical Center, University of Oslo, Norway

## Abstract

**Background:**

Hodgkin's lymphoma survivors (HLSs) commonly report chronic fatigue, defined as high levels of fatigue for 6 months or more. Underlying mechanisms are poorly understood. Based upon knowledge from other populations, lifestyle parameters may be related to this increased and persistent fatigue. The primary objective of the present study was to assess self-reported levels of physical activity, smoking habits and sleep patterns in HLSs with and without chronic fatigue. The secondary objective was to compare these results with data from age and gender adjusted data from the general population (Gen-Pop).

**Methods:**

The Fatigue Questionnaire (FQ) and questions about daily smoking, sleep patterns and level of physical activity were completed by 476 HLSs treated at Rikshospitalet-Radiumhospitalet Trust (RR). The Gen-Pop data was derived from 56.999 inhabitants in a Norwegian county responding to a mail survey. Fischer's exact test, chi square test and t-tests were used to compare groups. P-values < .05 were considered statistically significant. A logistic regression analysis was performed in comparing the Gen-Pop with the HLSs.

**Results:**

Level of physical activity, smoking habits and sleep patterns did not differ significantly between HLSs with and without chronic fatigue. The multivariate logistic regression analysis adjusting for different covariates, showed significantly more physically active men among HLSs compared with the Gen-Pop (OR = 1.50, CI 1.04 – 2.17), p = .031. No significant difference was found among females (OR = 1.20, CI = 0.83 – 1.74), p = .33.

**Conclusion:**

Lifestyle parameters did not seem to be related to increased and persistent fatigue among HLSs. The results may indicate that the experience of Hodgkin's lymphoma increases the level of physical activity among male HLSs.

## Background

Hodgkin's lymphoma (HL) is a disease mainly affecting young adults and with a good prognosis for survival since more than 80% of HL patients can expect to live free of disease five years after diagnosis [1]. Thus, HL generates a population of survivors with long life expectancies. Hodgkin's lymphoma survivors like other cancer survivors are at increased risk of late effects such as secondary malignancies, cardiac diseases, pulmonary and psychosocial problems [2-4]. In general, the health related quality of life of survivors are slightly reduced [5,6].

Fatigue in Hodgkin's lymphoma survivors (HLSs) has gained special attendance since it was first described in 1987 [7]. The experience of chronic fatigue (CF), defined as elevated levels of fatigue for more than 6 months, is significantly higher in HLSs than in the general population [8,9]. Studies comparing HLSs to testicular cancer survivors (TCSs) and to the general population have demonstrated that the prevalence of chronic fatigue is higher among HLSs (25–26%) than among TCSs (16%) and the general population (10–11%) [8-12]. However, there is limited knowledge about the underlying mechanisms of persistent fatigue in disease-free cancer patients after successful curative treatment. HLSs with pulmonary dysfunction report more fatigue than HLSs with normal pulmonary function [6]. HLSs with chronic fatigue also report decreased subjective physical functioning compared to non-fatigued survivors, and HLSs in general report decreased levels of physical functioning compared to the general population [5,8].

Acute fatigue most probably starts with a biological process that has ensued from the disease and/or its treatment. Most patients interpret fatigue as a signal to limit their activity and to rest. This may be effective in relation to acute fatigue in order to regulate the balance between rest and activity, resulting in restoration when needed. However, the experience of fatigue may provoke psychological and biological reactions that maintain or exacerbate fatigue in a vicious circle. Chronic fatigue, somatic sequelae and psychological distress separately or in combination may lead to decreased levels of physical activity. Theoretically, chronic fatigue may therefore lead to low level of physical activity in HLSs and subsequently intensify the experience of fatigue. To our knowledge, no investigators have evaluated this topic in detail in a large sample of HLSs.

Physical exercise has shown promising effects in terms of reducing the level of fatigue in patients with a diagnosis of chronic fatigue syndrome (CFS), in cancer patients suffering from fatigue during and immediately after active treatment, and in a small pilot study among nine HLSs suffering from chronic fatigue [13-17].

Generally, physically active individuals seem to have a healthier lifestyle than physically inactive individuals. They report lower alcohol consumption, less smoking, better sleep and presumably tackle stress better than inactive people [18]. Cancer survivors are at greater risk of co-morbid chronic conditions and death from non-cancer causes, and it is therefore reason to assume that a healthy lifestyle in terms of regular exercise and sleep patterns, smoking cessation and maintaining a healthy diet is particularly important in cancer survivors [19]. Physical activity habits among cancer survivors compared to non-cancer controls have been evaluated in a number of studies showing mixed results [20]. In one study, long-term survivors of testicular cancer reported to be more physically active than men in the general population [21]. Compared to a non-cancer comparison group, there were no evidence of differences between cancer survivors with various diagnosis and controls regarding levels physical activity and vegetable and fruit consumption [22]. However, the cancer survivors were significantly more likely to smoke, but neither groups met the American Society recommendations for smoking, eating fruits and vegetables, engaging in regular physical activity or maintaining a normal weight [22]. Blanchard et al. reported that compared to non-cancer controls, breast cancer survivors engage in as much physical activity as controls, but the groups differ in types of physical activity [23]. However, no study has explored the level of physical activity in HLSs. Because of the high prevalence of chronic fatigue and the late effects in HLSs, it is of special relevance to compare them to the general population with reference to morbidity and mortality.

In the present cross-sectional study we therefore addressed the question of whether lifestyle parameters such as physical activity, smoking and sleep patterns may be associated with chronic fatigue among HLSs. The main aim was to compare the level of physical activity (LPA) in HLSs suffering from chronic fatigue with the LPA in non-fatigued HLSs. We also explored smoking habits and sleep patterns in the same populations. The secondary aim was to compare lifestyle parameters in survivors with and without chronic fatigue to men and women in the same age range from the general population. Finally, we identified parameters that influenced physical activity. The hypothesis was that the experience of chronic fatigued among HLSs reduces the level of physical activity.

## Methods

### Study samples

Data from a study among HLSs treated at the Radiumhospitalet-Rikshospitalet Trust (RR) were compared to data from a large population survey conducted in the county of North-Trøndelag in Norway (Gen-Pop), serving as general population controls [24]. In both studies, individuals were contacted by mail, received self-report questionnaires and one written reminder if they did not respond after the first mailing.

#### Hodgkin's lymphoma survivors (HLSs)

The present study was launched in 2002 with the overall aim to assess the level and prevalence of fatigue [8], quality of life [25] and lifestyle in HLSs treated at RR in the period 1971–1997. Beginning in 1971, all patients with Hodgkin's lymphoma treated at the RR were registered in a Hodgkin's registry. Survivors in the present study were identified from this database.

Before 1980, the majority (92%) of Norwegian cancer patients in the age group between 15 and 39 years of age who were diagnosed with Hodgkin's lymphoma received treatment at the RR. The corresponding percentages in the age groups 40–59 years and above were 80% and 53% respectively [26]. After 1980, the treatment of Hodgkin's lymphoma gradually became more decentralised, although the RR still serves as the referral hospital for a health region that includes about 50% of the total population of Norway. The eligibility criteria for the present study included patients diagnosed for Hodgkin's lymphoma in the period 1971–1997, 15 years or older at the time of diagnosis and aged 20–74 years in 2002. All patients should be in complete remission, without signs of secondary cancers, and should not have received any treatment for Hodgkin's lymphoma during the last three years prior to assessment.

#### General Population (Gen-Pop)

From 1995 to 1997, all inhabitants in the county of North-Trøndelag in Norway aged 18 years and above were invited to participate in a large cross-sectional study of physical and psychosocial health. Individuals were asked to complete a questionnaire packet and to undergo a physical examination. The participation rate was 71%. The eligibility criteria for this study allowed for the inclusion of men and women who were between 20 and 74 years old when answering the questionnaire.

### Measures

The HLSs and the Gen-Pop received different self-report questionnaires. However, the variables included in the present analyses were identical in both cohorts. The questions are the same as those asked of participants in the North-Trøndelag health study (the HUNT study) in Norway and can be found in an article by Holmen et al. [24]. For the HLSs, the packet also included the Fatigue Questionnaire (FQ) for use in the present study [27]. Relevant clinical variables among the HLSs, such as date of diagnosis, stage, histology, treatment and current disease status (relapse or not) were retrieved from the Hodgkin's database at the RR.

#### Fatigue questionnaire (FQ)

The FQ (11 items) is intended to measure fatigue severity and to detect cases in clinical and epidemiological studies [27]. The FQ asks about fatigue symptoms experienced during the last month compared with how the subject felt when she or he was last feeling well. Additionally, two items ask about the duration and the extent of fatigue. Seven items measure physical fatigue (PF) and four items measure mental fatigue (MF). All 11 items are designated total fatigue (TF). Each item has four response choices. Likert-scoring (0, 1, 2, 3) is used for the construction of PF, MF and TF, with higher scores implying more fatigue. A dichotomised score (0, 0, 1, 1) is used in the definition of chronic fatigue; a sum of dichotomised scores ≥ 4 and a duration of six months or longer is designated chronic fatigue.

The FQ was originally validated in primary care, and has demonstrated good face and discriminant validity and good and stable psychometric properties across populations [27,28]. A two-dimensional structure (mental and physical fatigue) is confirmed in a norm study, and the two subscales correlated moderately (r = .46) [27]. Discriminant validity was further supported by the differences in TF between the different health status [27]. No specific validation study has been performed in cancer patients. However, the FQ has been use in studies among both Hodgkin's lymphoma patients and in patients with prostate cancer receiving hormonal therapy [28,29]. The psychometric properties demonstrated in these studies correspond with reports from the validation study and from studies in non-cancer populations. By comparing the FQ with the fatigue question in the Revised Clinical Interview Schedule, a relative operating characteristics analysis suggested that a cut off point of 4 or higher on a dichotomized scale would be the optimum cut off for a case definition.

#### Level of physical activity

**Figure 1:**
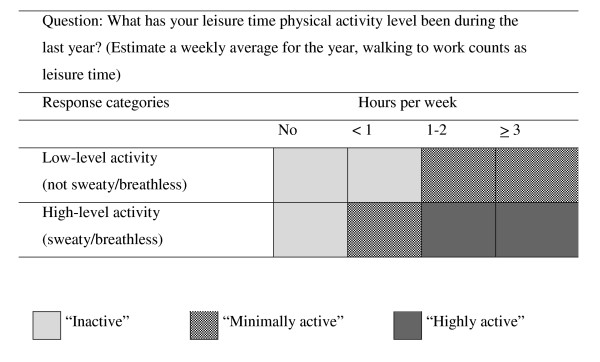
Level of physical activity (LPA).

The main study outcome was level of physical activity (LPA), and was assessed by one question, which described two sublevels of physical activity. The first described a low level of activity, such as walking, the other a high level of activity that leads to sweating and breathlessness. Forms with missing values for both responses were excluded from analyses. This question has recently been validated [30]. The question for hard LPA has acceptable repeatability and appears to be a valid measure for vigorous activity. The participants were divided into three groups, according to their level of physical activity (LPA) (see Figure 1) [21]:

**Group 1**. 'Inactive': no low-level activity or low-level activity < 1 h per week and no high-level activity.

**Group 2**. 'Minimally active': low-level activity ≥ 1 h per week, and either no high-level activity or < 1 h per week.

**Group 3**. 'Highly active': independent of the level of low-level activity, high-level activity ≥ 1 h per week.

Groups 2 and 3 were combined into one representing the physically active group and compared with group 1 (inactive group) in the logistic regression analysis.

#### Smoking and sleep patterns

Two questions were used to survey sleep patterns: 1) 'during the last month, have you had trouble falling asleep?' and 2) 'during the last month, did you wake up early and were unable to get back to sleep?' The response categories were: 'never', 'sometimes', 'often' and 'almost every night'. In the final analysis, the answers were dichotomised to a yes/no variable with never/sometimes categorised into 'no' (not having sleeping problems) and often/almost every night categorised into 'yes' (having sleeping problems) [26].

One question was used to map smoking habits: 'Do you smoke cigarettes or cigars daily?' with the response alternatives being yes/no [26].

### Statistical analysis

Comparisons between groups were performed by Fisher's exact test for nominal variables, chi square test for trend (linear-by-linear test) for ordinal variables, and t-tests for scale variables. P-values < .05 criteria were considered as statistically significant.

A logistic regression analysis with physical activity (yes versus no) as the dependent variable was performed in the analysis comparing the Gen-Pop with the HLSs, adjusting for the covariates age, group (HLSs/Gen-Pop) and educational level. The odds ratios (OR) are presented as estimates and 95% Confidence Intervals (95% CI). All tests were two-sided. The analysis was performed in the entire population (men and women) and men and women separately. Possible interactions were checked, as well as linearity in age. Significant interactions (only including gender) were accounted for by using separate analyses for men and women. All analyses were performed using SPSS for Windows (PC version 12.0).

### Ethical considerations

Data collection was conducted according to the guidelines of Helsinki Declaration. The Regional Committee for Medical Research Ethics, Health Region I, Norway, and the Institutional Review Board at the NRH approved the study. Appropriate informed consent was obtained from all respondents.

## Results

A total of 611 patients met the inclusion criteria. Ten patients were no longer registered with the Norwegian Census Bureau and could not be contacted. Ten patients who had received the majority of their treatment in another health region and who had participated in another postal survey on psychosocial late effects prior to the present were not contacted. The remaining 591 eligible patients were contacted by mail. A total of 479 returned the questionnaire packet. However, three patients failed to fill in the FQ, and were excluded from analysis in this report. Thus, the response rate for the FQ was 81% (476/591). The respondents had a mean age of 46 years (range 21–73) and 56% were males (*N *= 267).

There were more males than females among the non-responders (21% v 14.5%, respectively (p < .05), whereas no significant differences were found with age, observation time, primary treatment, and relapse between responders and non-responders.

One hundred forty-three of the 476 HLSs reported CF (30%). The basic characteristics among fatigued and non-fatigued HLSs are presented in Table 1. No significant differences in basic characteristics between the fatigued and the non-fatigued groups were found, except for stage/substage and a trend towards a shorter interval time since diagnosis among the fatigued survivors (204 versus 189 months, p = .07)

**Table 1:** Basic characteristics of fatigued and non-fatigued HLSs

	**HLSs with chronic fatigue (*N *= 143)**	**HLSs without chronic fatigue (*N *= 333)**	***p-value***
**Gender (N (%))**			.87
Male	81 (56)	186 (57)	
Female	62 (44)	147 (43)	
**Age at time of the study**			.37
(years)(mean ± SD)	47 (11.3)	46 (11.8)	
**Age at diagnosis**			.98
(years)(mean ± SD)	30 (10.6)	30 (10.6)	
**Observational time**			.07
(months)(mean ± SD)	204 (89.5)	189 (82.6)	
**Marital status (N (%))**			.47
Single	21 (14)	38 (11.)	
Married/cohabitant	105 (73)	250 (75)	
Divorced/separated	3 (2)	9 (3)	
Widow/widower	14 (10)	35 (11)	
**Educational Level (N (%))**			.11
≤ 10 years	29 (20)	60 (18)	
≥ 11 years,	54 (38)	151 (46)	
University < 4 years	21 (15)	67 (21)	
University ≥ 4 years	38 (27)	49 (15)	
**Stage/substage N (%)**			.05
IA/IIA	60 (42)	171 (51)	
IB/IIB	30 (21)	39 (12)	
IIIA/IVA	23 (16)	58 (17)	
IIIB/IVB	29 (20)	65 (20)	
**Primary treatment (N (%))**			.18
Chemotherapy	26 (18)	55 (16)	
Radiotherapy	39 (27)	108 (32)	
Radiotherapy +Chemotherapy	76 (53)	168 (51)	
Other treatment	1 (1)	1 (1)	
Missing	1	3	
**Relapse N (%)**	16 (1)	38 (11)	.81
Non-CR	11 (8)	23 (7)	

Self-reported physical activity level, smoking habits and sleep problems did not differ significantly between HLSs with and without fatigue (presented in Table 2). The logistic regression analysis with physical activity (yes/no), smoke (yes/no) and sleep disturbance (yes/no) as dependent variables adjusting for age, gender and level of education yielded similar results, with no differences in self-reported physical activity levels between chronic fatigued and non-fatigued HDS groups (results not shown).

**Table 2:** Comparison between HLSs with chronic fatigue and HLSs without chronic fatigue in self-reported physical activity, smoking habits and sleep patterns

	**HLSs with chronic fatigue (*N *= 143) (30%)**	**HLSs without chronic fatigue (*N *= 333) (70%)**	***p**-value*
**Smoker N (%)**	39 (28)	77 (24)	.38
Missing	5	17	
**Physical activity (N (%))**			.52
'Inactive'	22 (15)	55 (17)	
'Minimally active'	48 (34)	120 (36)	
'Highly active'	73 (51)	158 (47)	
**Problems to get to sleep (N (%))**			.49
'Never/sometimes'	98 (70)	243 (73)	
'Often/almost every night'	42 (30)	88 (27)	
Missing	3	2	
**Early wakening (N(%))**			.49
'Never/sometimes'	98 (70)	244 (74)	
'Often/almost every night'	42 (30)	87 (26)	
Missing	3	2	

**Figure 2:**
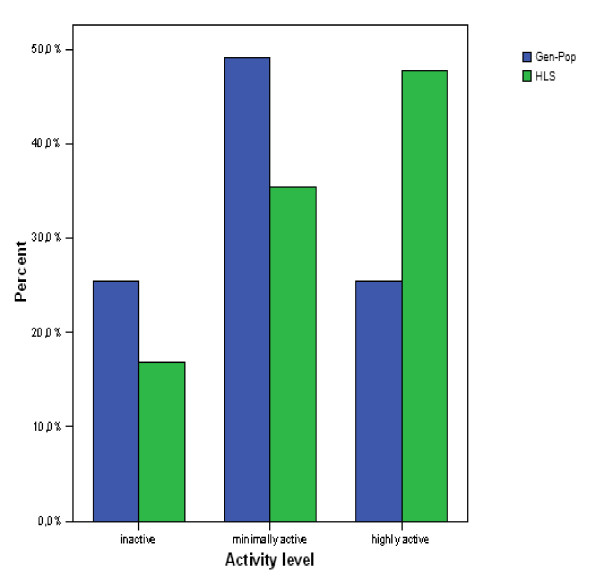
Comparisons between Hodgkin's lymphoma survivors (HLSs) and Gen-Pop in self-reported physical activity divided into three levels (inactive, minimally active and highly active).

The fatigued and the non-fatigued HLSs were therefore combined into one group (HLSs) and compared with the Gen-Pop in the subsequent analyses (Table 3). There were no significant differences in age across samples; however, the proportion of men was significantly higher in the HLSs sample, 56% versus 44% (*p <*0.0001). Among the HLSs, significantly more individuals had higher education, 37% versus 21% respectively (p < 0.0001). A significantly lower proportion of smokers was found in the HLSs compared to the Gen-Pop, 25% versus 31%, (p = 0.005). Furthermore, the HLSs reported to be more physically activey than the Gen-Pop, with 48% and 25% respectively being highly active, (p < 0.0001) (see Figure 2). In contrast, however, the HLSs reported significantly more problems than the Gen-Pop with falling asleep and waking up early (p < .0001).

**Table 3:** Comparison between HLSs and the Gen-Pop

	**HLSs **(*N *= 476)	**Gen-Pop **(N = 56999)	***p*-value**
**Age at questionnaire **(years)(mean ± SD)	46 (11.6)	47 (14.3)	.19
**Gender N (%)**			< .0001
Male	267 (56)	27082 (47)	
Female	209 (44)	29917 (53)	
**Smoker N (%)**	115 (25)	16916 (31)	.005
**Education**			< .0001
'< 10 years'	89 (19)	18453 (34)	
'> 11 years'	204 (44)	24611 (44)	
'University < 4 years'	88 (19)	7144 (13)	
'University ≥ 4 years'	87 (19)	4595 (8)	
**Physical activity (N (%))**			< .0001
'Inactive'	77 (16)	14510 (26)	
'Minimally active'	167 (35)	27973 (49)	
'Highly active'	231 (49)	14516 (25)	
**Problems to get to sleep (N (%))**			< .0001
'Never/sometimes'	341 (72)	43394 (92)	
'Often/almost every night'	130 (28)	3903 (8)	
**Early wakening (N(%))**			< .0001
'Never/sometimes'	342 (73)	43139 (91)	
'Often/almost every night'	129 (27)	4254 (9)	

A multivariate logistic regression analysis with physical activity (yes/no) as the dependent variable and sample (Gen-Pop/HLSs), age, education, smoking (yes/no) and early wakening (yes/no) as covariates was performed. Because of interaction, separate analyses were done for men and women. Being a male HLSs increased the chance of being physically active by 50% compared to males in the Gen-Pop, (OR = 1.50, CI 1.04 – 2.17), p = .031 (Table 4), while no significant difference was found among the females (OR = 1.20, CI = 0.83 – 1.74), p = .33, (Table 5). For both genders, physical activity (yes/no) increased with higher level of education, decreased with increasing age, and was lower among smokers than non-smokers. Finally physically active persons reported less sleep disturbances than inactive individuals.

**Table 4:** Multivariate logistic regression analysis among men with physical activity (yes/no) as the dependent variable

	**'Physical activity'**			
	No (0)	Yes (1)	OR (95%CI)	p-value

**All**				
**Gen-Pop **(0)	6367 (23)	20715 (77)	1.0	.031
**HLSs **(1)	36 (13)	231 (87)	1.50 (1.04 – 2.17)	
**Age **(years) (SD)	50 (14.6)	47 (14.0)	0.993 (0.991 – 0.996)	< .001
**Education**				
Primary/secondary school (0)	2516 (32)	5342 (68)	1.0	< .001
High school (1)	2706 (21)	10209 (79)	1.58 (1.47 – 1.72)	
College/university < 4 years (2)	416 (13)	2838 (87)	2.87 (2.51 – 3.29)	
College/university ≥ 4 years (3)	220 (9)	2157 (91)	3.81 (3.22 – 4.50)	
**Sleeping problems**				
No (0)	4520 (22)	16145 (78)	1.0	< .001
Yes (1)	397 (29)	974 (71)	0.74 (0.65 – 0.85)	
**Smoking**				
No (0)	3761 (21)	14468 (79)	1.0	< .001
Yes (1)	2214 (29)	5418 (71)	0.71 (0.66 – 0.77)	

**Table 5:** Multivariate logistic regression analysis among women with physical activity (yes/no) as the dependent variable

	**'Physical activity'**			
	No (0)	Yes (1)	OR (95%CI)	p-value

**All**	8184	21941		
**Gen-Pop **(0)	8143 (27)	21774 (73)	1.0	
**HLSs **(1)	41 (21)	167 (79)	1.20 (0.83 – 1.74)	.33
**Age **(years) (SD)	53 (14.6)	45 (13.7)	0.977 (0.975 – 0.979)	< .001
**Education**				
Primary/secondary school (0)	4026 (38)	6658 (62)	1.0	
High school (1)	2480 (21)	9420 (79)	1.64 (1.53 – 1.77)	
College/university < 4 years (2)	552 (14)	3426 (86)	2.49 (2.21 – 2.80)	< .001
College/university ≥ 4 years (3)	259 (11)	2046 (89)	3.16 (2.71 – 3.69)	
**Sleeping problems**				< .001
No (0)	5662 (24)	17408 (76)	1.0	
Yes (1)	956 (36)	1706 (64)	0.76 (0.69 – 0.83)	
**Smoking**				
No (0)	4797 (25)	14141 (75)	1.0	
Yes (1)	2801 (30)	6598 (70)	0.78 (0.69 – 0.83)	< .001

A multivariate logistic regression analysis with physical activity (yes/no) as the dependent variable and sample (Gen-Pop/HLSs), age, education, smoking (yes/no) and early wakening (yes/no) as covariates was performed using a different cut-off point between active and inactive. Groups 1 and 2 were combined into one representing the physically inactive group and compared with group 3 (active group) in the logistic regression analysis yielded same results among the males. Being male HLSs increased the chance of being active compared to males in the Gen-Pop, (OR = 2.2, CI 1.7 – 2.9), p ≤ 0.001. However, using this cut-off point, also the female HLSs increased the chance of being active compared to females in the Gen-Pop (OR = 2.6, CI 1.9 – 3.6), p ≤ 0.001 (results not presented in tables).

## Discussion

In this cross-sectional study, the level of self-reported physical activity and other self-reported life-style variables (i.e. smoking and sleep patterns) did not differ between chronic fatigued and non-fatigued HLSs. Furthermore, more male HLSs were physically active than in the general population, but no such difference was found among the females. Among both genders, those individuals who reported being physically active had a significantly higher level of education, were significantly younger, and were less likely to report smoking or having sleep problems.

The major strengths of this study are the comparison with the general population, the use of the same questionnaire to assess the level of physical activity, smoking and sleep in the two populations as well as the large sample sizes in both cohorts. The response rate was good and it is not likely that the non-responders have biased our results. The sample from the general population is representative because it includes all subjects in the North-Trøndelag county with same age range as the HLSs.

Self-report questionnaires are often applied to evaluate LPA in population based studies. However, all types of self-reporting tools are vulnerable to bias and self-report of physical activity habits is problematic in terms of validity. There is currently no standardised method for assessment of physical activity, and there is also no "gold standard" against which other methods can be validated. We regard self-report with two final response categories as sufficient for the purposes of this study. One recently published study showed that the use of different numerical response scales had a significant impact on the estimated percentage of regular exercisers [31]. Earlier studies have recommended a division of the level of physical activity into two main groups reflecting 'physically active' and 'physically inactive' individuals respectively. We therefore divided the level of physical activity into two main groups reflecting 'physically active' and 'inactive' individuals because this has been recommended by others [32]. Reliability and validity studies on self reported physical activity have shown that grouping physical activity into two categories (high intensity versus less than high intensity) resulted in a higher reliability and validity than setting the cut-off point between low and no activity [33].

However, we cannot exclude the possibility that people who have been seriously ill are especially aware of their health status and therefore report being more physically active than those without a history of cancer. Although there is a risk of misclassification of level of physical activity, its impact is estimated to be limited.

Our finding that fatigued and non-fatigued HLSs reported the same levels of physical activity was surprising. Exercise training is one of the few interventions suggested to prevent or alleviate fatigue among cancer patients and survivors, but so far the research supporting this suggestion and the mechanisms behind it are still limited [17,34-39]. In line with our findings that the physical activity level was equal in fatigued and non-fatigued survivors, no significant difference in peak exercise capacity (VO_2 _peak) was found between the fatigued and the non-fatigued HLSs in a previous pilot study [17]. However, the number of patients in this pilot study was small and a possible type II error cannot be ruled out.

Self-reported physical activity may give an indication of how much physical exercise/activity individuals undertake (categorised as being physically active or not active). However, complementary data on type, frequency and content of physical exercise/activity are necessary to be able to draw conclusions regarding possible interventions against chronic fatigue. Future studies should therefore be designed in order to thoroughly and objectively assess physical performance in chronic fatigued and non-fatigued cancer survivors. Possible approaches could involve measuring maximal oxygen capacity, muscle strength and/or monitoring physical activity with monitoring devices during the day [40].

The North-Trøndelag County population is considered to be representative of the Norwegian population as a whole. However, it is a rural district without densely populated areas and no cities with more than 50,000 residents. The level of education and income is lower than the national average. Low education and low income are shown to be associated with low levels of physical activity [41]. In accordance with these limitations, an extensive generalisation to Norway may be limited. A possible underreporting of the level of physical activity may also exist in this population. They may be physically active during work and daily activities, but may not perceive this as being physically active in terms of exercise. Our society and way of life have changed in the past few decades, and both daily activities and physical activity patterns have changed dramatically, while the need for physical strength and endurance has decreased. Studies report that daily physical activities have decreased, while exercise leading to sweating has increased during this period [42].

Results from studies comparing physical activity levels among cancer survivors to the general population are mixed. As compared to the general population, most studies suggest that cancer survivors have higher levels of activity, but some suggest no difference and one suggests less [20]. In support of our findings, Thorsen and colleagues recently reported similar results in testicular cancer survivors (TCSs). These individuals were significantly more physically active than the general population [21]. Among a group of lymphoma/leukaemia patients compared to non- cancer controls, there was a trend towards more controls being physically inactive than the cancer survivors [43]. Other recently published studies suggested that breast cancer survivors and a group of mixed cancer survivors were as physically active as healthy controls [23,43].

In our study, 25% of the HLSs reported being daily smokers, versus 31% in the Gen-Pop. As opposed to our findings, Ruffer et al. found a higher percentage of smokers among HLSs compared to a matched control group [9]. Unfortunately, they did not report on physical activity levels among their patients. A higher proportion of smokers among TCSs compared to healthy controls were also found by Thorsen et al. [21]. In contrast, Nord et al. found no significant difference in smoking habits when comparing a group of mixed cancer survivors to matched controls. However, a sub-analysis investigating only lymphoma/leukaemia patients showed results similar to those from our study, with a significantly lower percentage of smokers (13% versus 29%) [43].

Surviving a life-threatening disease, such as cancer, may lead to alterations of habits and life-style with more focus on health-promoting activities. Newly published results indicate that cancer survivors are likely to make lifestyle changes; however, those who are male, older and less educated are less likely to adopt these changes [20,44].

This is important, because the risk of developing late effects (caused by radiation and/or chemotherapy treatment) such as cardiovascular disease may be even higher than the risk of relapse or secondary malignancies [45-47]. The majority of patients who get the diagnosis Hodgkin's lymphoma are young men (< 40 years), and most are cured. Increasing physical activity has clearly been shown to attenuate cardiovascular risk in men, although other unfavourable coronary risk factors remained unchanged [48,49]. Because of this notable risk in Hodgkin's lymphoma survivors, it is important to obtain more insight in the level of physical activity and the physical fitness these patients have.

## Conclusion

Although a high proportion of HLSs suffer from chronic fatigue, they report the same levels of physical activity, smoking and sleep problems as non-fatigued individuals. Compared to a general population sample, the HLSs as a group smoke less, and a higher proportion of the HLSs are physically active. However, our results indicate that HLSs have more sleep problems. Given the present knowledge of the association between chronic fatigue and sleep problems, future studies should further explore a possible association between sleep disruptions and fatigue.

## Competing interests

The author(s) declare that they have no competing interests.

## Authors' contributions

LMO was responsible for collection of the data and writing the paper. SL was responsible for and conducted the data analysis in cooperation with LMO and LT. JHL, LMO, HH, ABJ, SK and SDF conceived and designed the study. MJH and JHL contributed to the writing of the paper together with the first author. All authors read and approved the final manuscript.

## Pre-publication history

The pre-publication history for this paper can be accessed here:


